# Psoriasis Treatments: Emerging Roles and Future Prospects of MicroRNAs

**DOI:** 10.3390/ncrna11010016

**Published:** 2025-02-13

**Authors:** Li Tian Keane Teo, Nerissa Juantuah-Kusi, Gowtham Subramanian, Prabha Sampath

**Affiliations:** 1 Department of Life Sciences, Imperial College London, Sir Ernst Chain Building, South Kensington, London SW7 2AZ, UK; 2 Division of Biomedical Sciences, Warwick Medical School, University of Warwick, Coventry CV4 7AL, UK; 3 A*STAR Skin Research Labs (A*SRL), Agency for Science, Technology, and Research (A*STAR), 8A Biomedical Grove #06-06 Immunos, Singapore 138648, Singapore; 4 Skin Research Institute of Singapore (SRIS), 11 Mandalay Road #17-01 Clinical Sciences Building, Singapore 308232, Singapore; 5 Program in Cancer and Stem Cell Biology, Duke-NUS Medical School 8 College Road, Singapore 169857, Singapore

**Keywords:** psoriasis, microRNAs, therapeutics, pathogenesis, inflammatory skin diseases

## Abstract

Psoriasis, a widespread and chronic inflammatory skin disorder, is marked by its persistence and the lack of a definitive cure. The pathogenesis of psoriasis is increasingly understood, with ongoing research highlighting the intricate interplay of genetic, immunological, and environmental factors. Recent advancements have illuminated the pivotal role of microRNAs in orchestrating complex processes in psoriasis and other hyperproliferative skin diseases. This narrative review highlights the emerging significance of miRNAs as key regulators in psoriasis pathogenesis and examines their potential as therapeutic targets. We discuss current treatment approaches and the promising future of miRNAs as next-generation therapeutic agents for this condition.

## 1. Introduction

Psoriasis is the most prevalent immune-mediated, non-communicable, chronic inflammatory papulosquamous skin disorder, with its causes and cure still shrouded in mystery. This lifelong condition affects individuals of all ages and is found across the globe, with reported prevalence rates ranging from 0.09% to 11.43% [1]. It is characterised by spontaneous remissions of compartmentalised erythematous plaques covered by white scales, the infiltration of inflammatory cells, and abnormal proliferation of keratinocytes [2], severely affecting patients’ quality of life [3,4]. Based on the Global Burden of Diseases 2019 data, approximately 5,000,000 cases have been reported in 2019 [5]. With its increasing prevalence and the concerning lack of treatment methods able to achieve remission, there is a need to address the disease.

The aetiology of psoriasis could be attributed to an interplay of genetic predisposition and environmental factors. Recent research strongly supports the central role T cells play in the development of psoriasis. This is evidenced by research showing that the majority of dermal inflammatory infiltrates in psoriatic lesions comprise activated CD4+ and CD8+ T cells [6]. These cells also express markers indicating memory status with varying stages of activation [7,8,9,10,11]. Additionally, the involvement of T cells in psoriasis is further substantiated by the efficacy of T cell-targeted therapies such as cyclosporine [12,13].

The pathogenesis of psoriasis involves the activation of T cells, their migration to the skin, and the release of pro-inflammatory cytokines. T cell activation is induced due to interactions with APCs followed by costimulatory signals that solidify the immune response [14,15,16,17,18,19,20]. After it has been activated, T cells migrate to the skin, driven by adhesion molecules such as CLA and selectins, resulting in inflammation [21,22,23].

Cytokines and their receptors act as communication links between the immune cells and keratinocytes, modulating the development of psoriasis. In psoriatic lesions, activated T cells predominantly release type 1 cytokines. This includes IL-2, IFN-γ, and TNF-α, which are responsible for sustaining the chronic inflammatory state whilst contributing to the psoriatic phenotype [24,25,26,27]. These mechanisms are crucial in driving the pathogenesis of psoriasis. Furthermore, cytokines like IL-17A and IL-23 are also key players in psoriasis, and therapeutic interventions targeting these cytokines and their receptors such as ustekinumab, guselkumab, tildrakizumab, and risankizumab presently provide relief to patients [28]. Moreover, psoriasis could be aggravated by environmental factors such as the weather, exposure to certain wavelengths of UV irradiation, the consumption of medication such as beta-blockers, lifestyle habits, and even microbial infections [29], which interact with immune-mediated mechanisms to influence disease pathogenesis. Although psoriasis is a multifactorial disease with various triggers, its immunological aspect and cytokine-mediated pathways are targets for therapeutic intervention.

This narrative review will discuss psoriasis, providing a brief overview of its pathogenesis and highlighting current treatment modalities, including topical, systemic, and other related therapies. It will further underscore the limitations of these existing options and the pressing need for novel therapeutic strategies. Furthermore, this paper will introduce the role of dysregulated miRNAs in psoriasis, examining their potential as both biomarkers and therapeutic targets, and explore how these small regulatory molecules can pave the way for future precision-based treatments.

## 2. Psoriasis—Characteristics and Symptoms

Psoriasis greatly impacts the quality of life of patients due to its inherent physical and psychological effects. The most common clinical form is psoriasis vulgaris or plaque psoriasis, which accounts for 85–90% of cases [30]. It primarily manifests as erythematous plaques marked with silvery scales, commonly appearing on the joint areas, scalp, lumbar areas, and the umbilicus region [31,32]. However, there are several other clinical forms of the disease that may manifest in different ways (Table 1)—(i) Psoriasis vulgaris, (ii) Guttate psoriasis, (iii) Inverse psoriasis, (iv) Erythrodermic psoriasis, and (v) Pustular psoriasis.

These various clinical forms of psoriasis present with unique physical manifestations and patterns. These forms are not only visually distinct but also vary in severity and therapeutic management. Hence, differentiating between the various clinical types is crucial for accurate diagnosis and treatment. Additionally, due to these manifestations, some of the common physical symptoms associated with psoriasis include itching, pain, or even bleeding. These symptoms may result in discomfort, sleep disturbances, or reduced mobility, which could make it difficult for patients to perform daily activities.

Psoriasis is also associated with systemic inflammation throughout the body, possibly leading to the development of comorbidities [33]. Some comorbidities that may arise due to psoriasis include cardiovascular disease, psoriatic arthritis, and other immune-modulated diseases such as inflammatory bowel disease [34]. This impacts the quality of life by affecting economic security and also the overall experience of life of these patients having to manage a plethora of illnesses. It is found that the increase in the economic burden of these comorbidities ranges from 30% to 194% of total medical costs with the use of current systemic treatments [35].

Furthermore, beyond the physical side effects of the disease, the psychological effects should not be underscored. Due to inherent stigmas, disfiguration as a result of psoriasis might trigger negative reactions from others or cause patients mental stress due to embarrassment or isolation. As a result, psoriasis is also associated with mental health issues such as depression, which is corroborated by a study by Dowlatshahi et al. showing that psoriatic patients were at a 1.5-time greater risk of having depression in comparison to controls [36].

Hence, compared to other major medical and psychiatric conditions, the physical and psychological impacts of psoriasis culminate in a disease that severely threatens the quality of life of patients with psoriasis, ranking above angina and hypertension in terms of detriment to life quality [37]. Currently, there are no definite cures for psoriasis, and all current available treatments simply aid in alleviating symptoms or clearing skin manifestations [32]. Furthermore, a large majority of the treatment methods implemented currently have varying and potentially toxic side effects.

With its debilitating effects on patients and increasing prevalence, there is a need to develop treatment methods that facilitate remission and prevent recurrence of the disease. This article aims to examine the advantages and limitations of current conventional therapies for managing psoriasis and explore how miRNAs can be leveraged as a potential therapeutic approach.

## 3. Current Treatment Options

The treatment of psoriasis is extremely complex. For instance, the presence of comorbidities can possibly complicate the achievement of remission. For example, in cases of hypertension or cardiovascular diseases, beta-adrenergic blocking agents are used to block the effects of epinephrine. These bind to the B1 and B2 receptors to inhibit renin release, a hormone that induces an increase in blood pressure and induces smooth muscle relaxation, respectively. This causes the heart to beat more slowly and with less force [38,39]. The mechanistic link is still not fully understood, but the use of beta-blockers may aggravate pre-existing psoriasis in patients or may even cause it to arise anew in those without psoriasis [40,41,42]. Therefore, a one-size-fits-all approach to psoriasis management is not feasible, and treatment strategies must be tailored to account for individual patient factors to ensure optimal outcomes.

Current treatments for psoriasis can be categorised into a few distinct classes:

Topical treatments (Table 2);

Systemic treatments (Table 3);

Alternative treatments (Table 4).

### 3.1. Topical Therapies

Topical therapies are used in the management of mild-to-moderate forms of the disease. They can be used as a monotherapy or as part of a combinatorial therapy with various systemic treatment methods (Table 2) [32,43,44]. Additionally, the use and application of such treatment methods are convenient and oftentimes are better tolerated in comparison to systemic treatment methods.

These treatment methods are typically applied directly to the skin and are often the preferred mode of therapy for patients with mild-to-moderate psoriasis. Some methods of treatment, such as coal tar or dithranol, are also essential, even in more severe cases, as they are used in conjunction with systemic treatments. However, each treatment varies in their potency, duration of effect, toxicity and patient tolerance. Hence, while effective, some treatments are limited by side effects such as skin irritation, staining, and impractical application, especially in long-term use.

### 3.2. Systemic Therapies

In more severe cases, which constitutes approximately 20 percent of psoriatic patients, systemic therapies (Table 3) become necessary, especially when the condition does not improve with topical treatments and significantly affects work or life or causes psychological discomfort. However, it is crucial to evaluate the therapeutic index of the treatment to ensure that the benefits of the treatment outweigh the potential risks [45].

These treatment methods are typically administered in a variety of ways. Each method has its respective advantages and is administered according to disease severity, affected body areas, and patient tolerance. However, with its significant side effects, treatment is typically reserved for more severe cases, particularly those that affect large areas of the body or greatly reduce quality of life. The greater side effect profiles of systemic therapies make them more suitable for patients with extensive or hard-to-treat psoriasis, as the risks of side effects are balanced against the need to alleviate severe symptoms. These methods facilitate effective and appropriate management of psoriasis at the respective levels of disease severity. 

Alternative treatments to the conventional tropical and systemic therapies have also been discussed and implemented. These alternative treatments highlighted in Table 4, all employing natural resources such as sun exposure and highly concentrated salt water, were shown to decrease the PASI scores of patients.

## 4. Emerging Therapeutics in the Pipeline

Due to the limitations of conventional treatment methods currently, it is an ongoing endeavour to develop therapeutics that provide greater efficacy and safety. Novel therapies are being used to target key molecular and immune pathways implicated in psoriasis. Table 5 highlights several promising therapies being developed for use in psoriasis management, detailing their mechanisms of action and target pathways.

This pipeline reflects the dynamic landscape of psoriasis treatment and how the landscape of psoriasis treatment is rapidly evolving. Many innovative therapies are being developed to target specific inflammatory pathways and immune responses involved in the pathogenesis of the disease.

These novel approaches aim to address the underlying mechanisms of psoriasis more precisely. This would enhance efficacy and reduce side effects associated with current treatment methods. These novel therapies could potentially offer higher-potency treatment with significantly fewer drawbacks.

## 5. Non-Coding RNA as a New Therapeutic Avenue

Recent advancements in molecular biology have highlighted the significant role of ncRNAs, specifically miRNAs, in the regulation of gene expression associated with psoriasis. miRNAs are endogenous, small, non-coding RNA molecules that typically range from 20 to 24 nucleotides in length. These molecules are crucial to gene regulation, functioning as post transcriptional regulators by targeting mRNAs for cleavage or translational repression [46,47].

miRNAs regulate the expression of at least a third of all human genes and over 60% of human protein-coding genes [48,49,50]. Differential expression levels of miRNA between healthy skin and psoriatic lesions demonstrates their involvement in the pathogenesis of psoriasis. Jiang et al. identified 251 miRNAs that were differentially expressed between healthy controls and patients with psoriasis through RNA-sequencing analysis [48]. These differentially expressed miRNAs are involved in various different pathways—neural regulation, hormone signalling, metabolic regulation, and cellular secondary messenger signalling [51]. Collectively, all these pathways contribute to the pathogenesis of psoriasis, whereby keratinocytes undergo hyperproliferation, abnormal differentiation, and the atypical activation of immune cells, which lead to inflammation of the epidermis and its associated comorbidities [52,53,54,55,56,57].

The therapeutic potential of miRNAs thus lies in their role as central regulators of these pathways. Regulating miRNA activity has emerged as a promising approach to target crucial pathological pathways in various diseases, opening up the possibility of application in psoriasis. Integrating miRNAs into the therapeutic landscape aligns with the growing understanding of the interplay between environmental and genetic factors in psoriasis pathogenesis, offering a novel avenue to potentially enhance or synergise with current biologic therapies.

The synthesis of mature miRNA begins in the nucleus, where RNA polymerase II or III carries out transcription to form Pri-miRNA [58]. Pri-miRNA is then modified by RNaseIII Drosha cleavage, resulting in the intermediary Pre-miRNA. Pre-miRNA is between 80 and 100 nucleotides long on average [59]. High Ran-GTP levels then drive the export of pre-miRNA from the nucleus into the cytoplasm via exportin-5 [60,61]. The pre-miRNA is then further processed by Dicer and RNase III, where it is cleaved, resulting in a 22-nucleotide mature duplex miRNA. Within the duplex, the strand with the less thermodynamically stable 5′ end is deemed the guide strand, whilst the other strand is the passenger strand. When the duplex is subsequently unwound, the passenger strand is degraded. The guide strand is then associated with an Ago protein, which plays a key role in mediating RNA silencing [62]. The mature miRNA forms a complex with a RISC to form a miRISC. The complex can then trigger Ago-catalysed endonucleolytic cleavage of target mRNA, resulting in mRNA degradation or the repression of translation [63,64,65].

### 5.1. miRNA Interactions in Psoriasis

**Figure 1:**
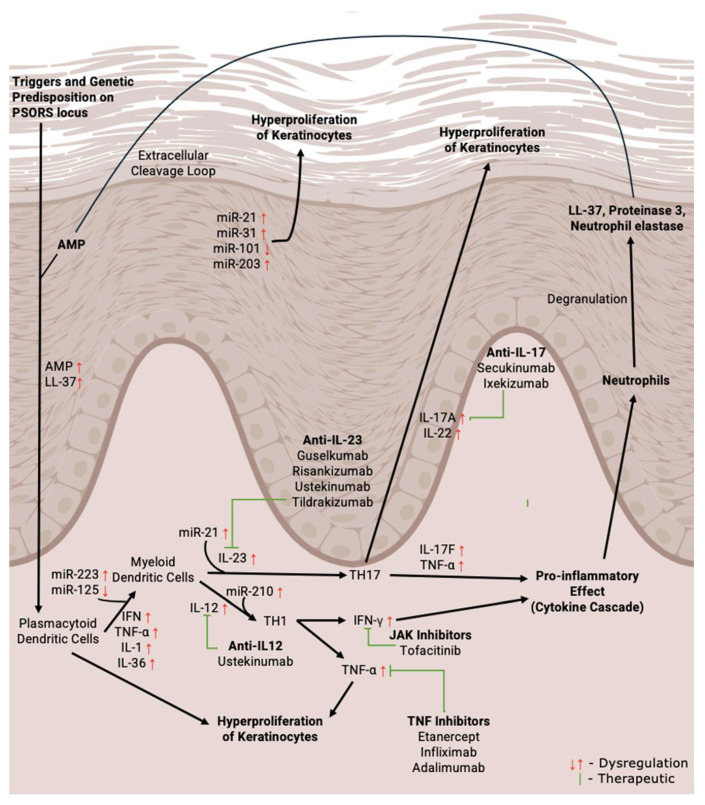
Role of miRNA in the pathogenesis of psoriasis with a comparative view of conventional therapeutic pathways. Various triggers such as environmental infections or irritants initiate a cascade of immune responses, causing the chronic inflammation seen in psoriatic lesions [66]. Genetic factors also contribute to the onset of psoriasis. The PSORS1 gene located on chromosome 6p21 is strongly associated with an increased risk of developing psoriasis. This gene affects immune function, which plays a role in regulating keratinocyte responses and dendritic cell activity [67]. When triggered, keratinocytes release AMPs. LL-37, a crucial AMP, forms complexes with self-DNA. This activates pDCs through the TLR9, which induces the production of type I interferons that initiate an immune response, exacerbating inflammation [68]. mDCs and pDCs then sustain the inflammatory response in psoriasis. Dysregulated miRNAs such as miR-223 and miR-125 influence dendritic cell maturation and activity, enhancing cytokine production and amplifying inflammation [69,70,71,72,73]. This enhances cytokine production of IL-12 and IL-23, which promotes the differentiation of naive T cells into TH1 and TH17 cells [74,75]. TH1 cells produces IFN-γ, activating keratinocytes and contributing to inflammation. Both TH17 cells secrete IL-22 and IL-17, which promotes keratinocyte proliferation and further inflammatory responses [76]. miR-21, miR-31, and miR-203 play key roles, with miR-21 suppressing apoptosis and promoting inflammation, while miR-31, miR-101, and miR-203 drive keratinocyte hyperproliferation by altering cell cycle regulation [48,77]. Upregulated pro-inflammatory cytokines like TNF-α, IL-1β, IL-6, and IL-8 further amplify the inflammatory response and drive keratinocyte proliferation [74]. Similarly, neutrophils release proteolytic enzymes during degranulation, which targets keratinocytes, releasing AMPs and further contributing to the inflammatory response. miRNA dysregulation (e.g., miR-223) also impacts neutrophil function, enhancing their role in disease progression [75,78,79]. Up and down arrows in the figure indicate the increases and decreased expression of the candidates.

The full spectrum of miRNA interactions and involvement in psoriasis is still not fully elucidated; however, the mechanisms of several validated miRNAs, as seen in Figure 1, can elucidate the function of miRNAs and how miRNAs can be potentially targeted in psoriasis. Biologic treatment methods have also been highlighted to provide a comparative view of their mechanism of action.

Immune cells in psoriatic patients release inflammatory cytokines, resulting in NF-κB activation, which is responsible for inducing the transcription of miR-31. miR-31 is involved in modulating chemokines and cytokine synthesis in keratinocytes by targeting a negative regulator of G1 to S phase ppp6c [53,80,81]. The upregulated miR-31 stimulates hyperproliferation of keratinocytes (as seen in Figure 1) and also the increased production of inflammatory mediators and leukocyte chemoattractants (e.g., TNF-α), contributing to psoriatic lesion formation [77].

Leukocyte chemoattractants, such as TNF-α, play a vital role in the immunopathogenesis of psoriasis. The expression of TIMP-3, involved in the inhibition of TACE, is reduced in the keratinocytes of psoriatic patients due to inhibition by upregulated miR-21 in psoriatic lesions. This results in an increase in levels of soluble TNF-α release, which causes skin inflammation [82,83]. This also causes a cascade to occur, in which elevated levels of soluble TNF-α binds to the TNF-α receptors, inducing the phosphorylation of STAT3. This results in the movement of STAT3 dimers into the nuclei, leading to a greater rate of transcription of miR-21 [77]. As such, levels of miR-21 are found to be significantly upregulated in the lesions of psoriatic patients [83,84,85]. Additionally, there are other molecular mechanisms that contribute to the elevated levels of miR-21 in psoriatic patients, such as elevated levels of TGF-β in psoriatic patients, inducing further transcription of miR-21 [83]. miR-21 inhibits the translation of SMAD7, a key molecule that inhibits TGF-β signalling as part of a negative feedback regulation. This sustains the high transcriptional rate of miR-21 as elevated levels of TGF-β are being sustained [86,87]. The mechanism of miR-21 was substantiated by Henriet et al., who showed that silymarin, a medicinal plant extract, ameliorates the severity of psoriasis by inhibiting the proliferation and differentiation of keratinocytes via modulation of miR-21/TGF-β pathway [88].

Similarly, miR-203 is upregulated in psoriatic patients. It modulates the cell cycle of keratinocytes—namely proliferation, differentiation, and apoptosis—and functions by targeting several epidermal genes [81]. miR-203 binds to the 3′ UTR region of p63 mRNA, a member of the p53 family. This halts the cell cycle at the G0/G1 stage and stimulates the abnormal keratinocyte differentiation characteristic of psoriasis [89,90,91]. Another target gene of miR-203 is the SOCS-3, which is also involved in regulating the proliferation and differentiation of keratinocytes [92]. Upregulated miR-203 plays a significant role in the development of psoriatic lesions, as it triggers uncontrolled hyperproliferation of keratinocytes through various different pathways, and this presents a potential therapeutic target in the treatment of psoriasis.

Alongside miR-21, miR-101 is also another key regulator of signalling molecules controlling keratinocyte differentiation, proliferation, and inflammatory responses. miR-101 is responsible for regulating keratinocyte differentiation and apoptosis; however, this balance between cell proliferation and shedding is disrupted in psoriasis [93]. A study by Quah et al. found that the notable downregulation of miR-101 in psoriatic lesions correlates with an upregulation of EZH2 [94]. EZH2 is a histone methyltransferase that causes hyperproliferation of keratinocytes and poor differentiation characteristics in psoriatic lesions. miR-101, which is a negative regulator of EZH2, binds to its mRNA and limits EZH2 expression [95,96]. However, due to the downregulation of miR-101, this drives unregulated EZH2 expression, which directly contributes to the hyperproliferative profile of keratinocytes observed in psoriasis.

Further research has also shown that IL-17 indirectly suppresses miR-101. IL-17 upregulates the lncRNA SNHG6, which sequesters miR-101 and reduces the suppressive effect on EZH2 expression. This IL-17/SNHG6/miR-101/EZH2 axis suggests that downregulation of miR-101 is part of a broader inflammatory pathway promoting the hyperproliferation of keratinocytes [97]. These various pathways illustrate the extensive role miRNAs have in psoriasis pathogenesis.

Beer et al. demonstrated that the miR-155 is upregulated in psoriasis lesional skin, with dysregulated miR-155 contributing to altered keratinocyte differentiation [98]. Several other studies have also shown the increased expression of miR-155 [99,100] and miR-210 in psoriasis [101]. However, further detailed mechanistic studies are required to establish the precise role of these miRNAs in disease progression. Chen and colleagues confirmed that the upregulation of miR-155 leads to the development of psoriasis by inducing the inflammatory response through the NF-κB pathway [102]. Several other miRNAs have been implicated in psoriasis, including miR-31 [53,80], miR-146a [103,104,105], and miR-203 [92,106,107,108], which are upregulated, and miR-125 [109,110], miR-99 [111,112,113], miR-197 [114,115], and miR-520 [116], which are downregulated in the skin and serum of patients with psoriasis. Collectively, these research works underscore the potential of miRNAs as biomarkers and therapeutic targets in disease manifestation. However, the variability in study designs and patient cohorts highlights the need for rigorous, cohort-based, and tightly controlled clinical studies to further validate the expression of these dysregulated miRNAs. These tightly regulated studies would provide greater clarity on the functional roles of these miRNAs in disease progression and pathogenesis and pave the way for the development of miRNA-based therapeutic strategies for psoriasis [117].

### 5.2. Targeting miRNAs in Psoriasis

miRNAs have been evidenced to be critical regulators of gene expression in psoriasis. Thus, this potentially opens up new avenues of novel therapeutic targets to modulate the inflammatory pathways and keratinocyte dysfunction central to disease pathogenesis. Other medical fields have begun exploring miRNA-based therapies, such as oncology, where Sayed et al. reviewed the role miRNAs played in tumorigenesis and metastasis, in addition to the potential and advancements of miRNA as a therapeutic [118]. Thus, based on differential expression levels of miRNAs between healthy samples and psoriatic patients, we can target specific aberrantly expressed miRNAs as an alternative strategy to alleviate psoriasis. Furthermore, miRNAs can also serve as prognostic/diagnostic biomarkers [119,120,121,122].

A key characteristic of miRNAs in serum is their remarkable resistance to degradation, even when exposed to circulating ribonucleases or extreme conditions such as prolonged storage at freezing temperatures or exposure to extreme pH levels [123,124]. Additionally, even in small-volume blood samples, miRNA can be readily detected via quantitative real-time PCR (qRT-PCR). With miRNA expression in psoriatic lesions being highly differential and their ease of detection, they can potentially be used as a biomarker either for diagnosis or as an indicator of therapeutic response [125]. Løvendorf et al. investigated the use of miR-223 and miR-143 as systemic biomarkers in psoriasis. In the study, miR-223 and miR-143 was found to be significantly upregulated in the peripheral blood mononuclear cells (PBMCs) of psoriatic patients in comparison to healthy samples. miR-223 and miR-143 expression levels strongly correlate with the PASI score [126]. This could become an alternative to the inherently limited PASI score used to determine the severity of disease. For instance, in patients with darker skin tones, erythema may appear to be more subtle or perhaps hyperpigmented. This could result in a lower overall PASI score and in turn deny the timely and effective management of the disease in such patients [127]. Hence, the PASI score, which relies on the ability to recognise and diagnose the disease, is limited by the experience and knowledge of clinicians. Using miRNAs as a biomarker could thus provide a more consistent and objective method in the quantification of the severity of psoriasis in patients. Furthermore, receiver operating characteristic (ROC) analysis has also further substantiated that expression levels of miR-223 and miR-143 can be used as biomarkers to differentiate between normal and psoriatic samples [126]. Tsuru et al. revealed that within the hair shaft at the non-lesional occiput of psoriatic patients, miR-424 was significantly upregulated in comparison to healthy samples [128]. This signifies that miRNAs have differing levels of expression in different parts of the body. This highlights that potential non-invasive sampling methods, such as hair, could be used as objective biomarkers in the diagnosis of psoriasis.

In terms of therapy response, studies by Løvendorf et al. also found that in psoriatic patients that underwent a course of treatment with methotrexate, these patients not only experienced a decrease in the severity of their psoriasis but also showed a significant downregulation of previously elevated levels of miR-223 and miR-143 [126]. Furthermore, studies by Xue et al. found that miR-205 was downregulated in the skin of psoriatic patients [129]. A study by Mihu et al. then subsequently discovered that miR-205 was upregulated in psoriatic patients that were treated in comparison to a control group that was not treated, with the difference being statistically significant [130]. As such, other than being used for diagnosis, miRNAs can alternatively be used as biomarkers to measure the therapeutic response of psoriasis treatments. Primarily, it can facilitate the evaluation of treatment efficacy to aid clinicians in determining whether a treatment plan is beneficial. It can also serve as a guide for clinicians to modify treatment plans through optimising dosages or switching to alternative therapies. Hence, miRNAs can serve to predict patient outcomes, minimise possible side effects, and improve overall disease management with real-time feedback on the success of the intervention.

Beyond their role as biomarkers, miRNAs also offer promising therapeutic potential in the treatment of psoriasis. In particular, by targeting and inhibiting miRNAs that are upregulated in psoriasis, such as those implicated in the inflammatory processes of the disease, it might unlock new avenues for treatment that could modulate the underlying mechanisms driving psoriasis. For instance, therapeutic inhibition using locked nucleic acid (LNA)-anti-miR on miR-21 has been studied by Javanmard et al. in the context of melanoma. In the study, miR-21 had an 80% reduction in its expression level just 24 h after transfection [131]. This underscores the potential of LNA-anti-miR technology in targeting upregulated miRNA with high specificity. Although the study was targeted at melanoma cancer cells, the successful inhibition of upregulated miRNAs provides a foundational proof of concept for a similar approach to be applied in psoriasis, where many miRNAs are also known to be overexpressed. miR-21 is a promising therapeutic target in psoriasis, as it plays a key role in regulating the proliferation and apoptosis of keratinocytes in psoriatic lesions. miR-21 exerts its function by binding to caspase-8 mRNA and silencing caspase-8 expression [132]. This reduces apoptosis in keratinocytes, thus promoting keratinocyte survival and hyperproliferation. miR-21 also inhibits TIMP-3 expression, resulting in the increased release of TNF-α, exacerbating the immune response in psoriasis [48]. As such, by effectively reducing miR-21 levels using LNA-anti-miR designed to bind specifically, the LNA-anti-miR prevents the interaction of miR-21 with its target mRNAs, such as that of caspase-8, and the inhibition of TIMP-3 expression. This could possibly rebalance the inflammatory, proliferation, and apoptosis levels of keratinocytes. This highlights the potential for miRNA to be targeted, as it can selectively modulate pathogenesis pathways in psoriasis whilst minimising systemic side-effects. However, the physiological context of psoriasis differs significantly from that of melanoma, especially in terms of cellular microenvironment, immune response, and the role miRNAs play in disease progression. Hence, the translation applicability remains speculative until similar studies are performed in keratinocytes or psoriasis-relevant models.

At the same time, while the expression of some miRNAs is upregulated, the expression of other miRNAs is downregulated in psoriatic lesions instead. Thus, while the inhibition of certain miRNAs offers therapeutic potential, elevating expression levels of downregulated miRNAs presents another promising approach in the treatment of psoriasis. It might even be possible to restore normal cellular functions and counteract the pathological processes driving the disease. miRNA replacement has been a therapeutic method studied as of recently, and this involves the delivery of miRNA mimics to restore the previously higher levels of miRNA present in healthy skin. A benefit of using miRNA mimics in replacement therapy is that they share an identical sequence as the naturally occurring miRNAs that are depleted in psoriatic lesions. This is crucial, as the miRNA mimics must function like naturally occurring miRNA, with specific miRNA–mRNA interactions, whilst also reducing the likelihood of non-specific off-target effects [133].

miR-125b is present in almost all organs and particularly in most skin types such as fibroblasts, keratinocytes, and melanocytes of the skin [92]. miR-125b is a highly downregulated miRNA in psoriatic lesions involved in repressing FGFR2 expression [109]. This dysregulation leads to the overexpression of FGFR2, thereby contributing to aberrant differentiation of keratinocytes and hyperproliferation. Additionally, miR-125b also regulates inflammatory cytokines such as STAT3 and TNF-α, crucial to the inflammatory cascade in psoriasis. Its deficiency elevates the levels of these cytokines, which amplify the inflammatory response, exacerbating the disease.

To address the dysregulation, Han et al. studied the topical delivery of a miR-125b mimic using framework nucleic acids (FNAs) to elevate the level of miR-125b [110]. Increasing the expression of miR-125b could inhibit unchecked keratinocyte proliferation whilst upregulating several markers of differentiation [109]. This could potentially arrest the hyperproliferation and abnormal differentiation that drives erythema formation in psoriatic patients. Han et al. synthesised the mimic by first forming the FNA through combining four single-stranded DNAs (S1, S2, S3, and S4), followed by annealing through heating and rapid cooling. Thereafter, the S2 was replaced with a S2-sticky end on the FNA to form FNA-ST, followed by annealing of a miR-125b mimic on FNA-ST to synthesise FNA-miR-125b. The FNA-miR-125b mimic was then incorporated into an oil-based cream for topical application. This was found to effectively manage the disease, as keratinocytes had reduced levels of keratinocyte proliferation whilst also having reduced levels of inflammatory cytokines present. FNA-miR-125b can be utilised to inhibit cell proliferation through the effective knockdown of downstream target mRNAs such as STAT3 and TNF-α, with its application method significantly improving skin penetration efficiency [110].

Hence, the therapeutic potential of miRNA replacement, particularly in restoring the expression of downregulated miRNAs such as miR-125b in psoriatic lesions, presents a compelling case to target miRNA in the management of psoriasis. The therapy demonstrates that it can effectively counter the hyperproliferation and abnormal differentiation occurring in the keratinocytes of psoriatic patients that arises due to downregulated miR-125b levels. The subsequent effective knockdown of pro-inflammatory mRNAs like STAT3 and TNF-α, coupled with enhanced skin penetration efficiency, are statistically significant in comparison to controls, which highlights the potential of significantly improving patient outcomes. However, even though the findings are promising, we must evaluate the robustness and clinical applicability of the methods. Han et al. had a well-structured methodology encompassing the assembly and delivery of FNA-miR-125b constructs [110]. The FNA assembly had enhanced stability and improved skin penetration efficiency, critical for therapeutic delivery [110]. While the results provide proof of concept, the use of a mouse model cannot fully replicate the pathophysiology of psoriasis in humans. Subsequent validation using human keratinocyte cultures for instance would substantiate the translational potential of the findings. Additionally, the long-term effects of this therapeutic approach have not yet been explored. It remains unclear whether treatment would result in sustained remission or whether symptoms would recur if treatment were discontinued. Furthermore, its relative efficacy is also unknown, as the miR-125b therapy was not directly compared with conventional treatments such as biologics or topical corticosteroids. This information is crucial to ascertaining the safety and feasibility of translating this therapy into a clinical setting. 

Similarly, miR-101 is significantly downregulated in psoriatic lesions. Its downregulation results in the derepression of SNHG6, a lncRNA that promotes the activity of histone methyltransferase, EZH2. EZH2 is known to drive keratinocyte hyperplasia, thus contributing to keratinocyte hyperproliferation. By restoring miR-101 levels, this could suppress the expression of SNHG6 to consequently reduce EZH2-mediated hyperplasia. As this mechanism bypasses the upstream IL-17 signalling pathway, this therapeutic approach could offer targeted treatment, in contrast to conventionally used IL-17 inhibitors, which can potentially cause immune suppression [134,135]. Additionally, miR-101 regulation of EZH2 also highlights its potential in mitigating keratinocyte proliferation without inducing significant cytotoxicity [136]. This further highlights the finding that therapeutic modulation of miRNA levels could serve as a novel, targeted treatment with minimal side effects and greater efficacy in managing psoriasis.

These outcomes suggest that miRNA replacement therapy could potentially address multiple aspects of psoriasis pathogenesis simultaneously, thus making it an extremely promising strategy. However, beyond the advantages, there might be some challenges to consider. Challenges might include, but are not limited to, maintaining the stability of miRNA to ensuring that sustained therapeutic effects are critical factors that need to be addressed. Additionally, the specificity of miRNA mimics is beneficial, as they would target specific mRNAs similar to their natural occurring counterparts, reducing the risk of off-target effects. However, this would still require optimised delivery mechanisms for consistent therapeutic outcomes.

Additionally, miRNAs are conserved in different species, and this allows for pre-clinical evaluation in animal models [125,137]. For example, a study by Wu et al. utilised topically applied imiquimod (IMQ), which induces psoriasis-like dermatitis in a murine model [138] and is built upon previous studies that highlighted elevated levels of miR-210 in psoriatic CD4+ T cells and PBMCs of patients with psoriasis in comparison to healthy controls [139]. Wu et al. further demonstrated that either the genetic knockout of miR-210 or the silencing of miR-210 by an intradermal injection of antagomir-210, a miR-210 inhibitor resulted in the prevention of psoriasis-like dermatitis in the IMQ model [140]. This was observed in both clinical manifestations and pathologically, with naïve CD4+ T cell differentiation towards Th1 and Th17 cells being less pronounced in the KO mutant mice or the antagomir-210-treated mice as compared to the WT mice [140]. These results indicated that miR-210 mediated the development of psoriasis via T cell differentiation and could present another target for therapeutic treatments.

The findings highlight the potential of targeting miRNAs as a therapeutic strategy for psoriasis. However, many of these miRNAs that could be promising targets for psoriasis treatments also carry with them potential implications in other cellular pathways and therefore side effects that could be serious and must be considered. Due to their role as regulatory factors of gene expression, many miRNAs have been connected to other biological disorders, which could present potential negative side effects in treatment. Previously reviewed by Sayed et al. [118], specific miRNAs can be involved in tumorigenesis alongside cancer progression [141], including some of the ones that have previously been discussed in this review. For example, miR-210 expression was reported to be down-regulated during epithelial–mesenchymal transition [142], the biochemical transformation process by which a polarised epithelial cell assumes a mesenchymal cell phenotype [143]. This enhances the migratory capacity of the cell and elevates resistance to apoptosis [144]. As carcinomas are derived from epithelial cells, a study by Tsuchiya et al. sought to investigate the functional role of miR-210 in the growth of carcinomas in both clinical samples and oesophageal squamous-cell carcinoma (ESCC) cell lines [145]. The results of this study suggested that miR-210 expression is not only down-regulated in cancerous ESCC cell lines and clinical samples but that the levels of miR-210 expression seemingly aligned with the degree of tumour differentiation [145]. Tsuchiya et al. thus suggested that miR-210 functioned as a tumour-suppressive miRNA in ESCC [145]. This occurs through the targeted suppression of fibroblast growth factor receptor-like 1 (FBFRL1). Similarly, a study by Huang et al. reported that miR-210 suppressed tumour growth in pancreatic tumour cell lines [146]. However, this is not the same for all cancers, with multiple studies identifying miR-210 as a major miRNA induced under hypoxia and frequently upregulated in cancers such as triple-negative breast cancer, with the levels of overexpression correlating with the aggressiveness of the cancer [147]. As such, despite the potential of miRNA as therapeutic targets in the treatment of psoriasis, there is also potential for serious side effects due to the involvement of the same miRNAs in other cellular pathways and disorders that must also be considered.

The various approaches not only hold promise for restoring normal cellular functions but also offer a targeted method to address the underlying mechanisms of the disease. However, overcoming the current limitations and challenges with reference to targeted delivery is essential to realise the benefits of this therapeutic approach in a clinical setting.

## 6. Conclusions and Future Directions

Psoriasis is a complex and multifaceted disease. Current therapies primarily manage symptoms rather than targeting its pathogenesis. Although current treatments provide certain amount of relief to the patients, their side effects and limitations call for the need to explore alternative approaches. The novel therapies highlighted in this review, including phototherapy-activated agents, gut-restricted immunomodulators, targeted monoclonal antibodies, and multi-action topical creams, highlight significant advancements in the management of psoriasis. However, despite the promising outcomes observed thus far, extended research is required to ensure the long-term safety and efficacy of these novel treatments.

Furthermore, advances in understanding the molecular mechanisms driving psoriasis have highlighted the extensive role miRNAs play in the pathogenesis of the disease. Specifically, miR-21, a well-studied miRNA in psoriasis, has been found to be implicated in the regulation of immune cell proliferation and the production of inflammatory cytokines. Hence, targeting miRNAs such as miR-21 could potentially develop therapies that go beyond symptomatic relief. Future studies focusing on combinatorial therapeutics with miRNA therapeutics, emerging biologic and small-molecule therapies, may be more effective as holistic treatment options with improved patient outcomes. For instance, phototherapy has been used in combination with some topical (vitamin derivatives) and systemic therapies (methotrexate) for the treatment of palmoplantar psoriasis. Very little evidence is available on the use of combinatorial therapies involving two biologics or a biologic and small molecules. More robust and tightly controlled clinical studies are warranted to establish the effectiveness and health benefits of using combinatorial therapies in psoriasis.

**Table 1:** Clinical manifestations of psoriasis and their distinct physical characteristics.

	Physical Manifestation
Psoriasis Vulgaris	Well-demarcated erythematous plaques covered with silvery-white scales. Commonly found on extensor surfaces [31,32]
Guttate psoriasis	Round or teardrop shaped (red, purple, or brown). Lesions are small and drop-like [148]. Plaques present on extremities and trunk.
Inverse psoriasis	Erythematous patches are smooth and shiny and can be found in skin folds, such as the anogenital, inframammary, and axillary areas [149].
Erythrodermic psoriasis	Widespread erythema that covers at least 75 percent of the skin. Less prominent scaling is observed in this form [150].
Pustular psoriasis	Two main forms: 1. Palmoplantar—this affects the palms and soles, and 2. Generalised—characterised by sudden eruptions of pustules on erythematous areas that cover much of the body [32,151].

**Table 2:** Overview of topical treatments in the management of psoriasis.

Type of Therapy	Description and Mechanism of Action	Side Effects or Disadvantages
Emollients	Typically composed of paraffin or aqueous creams to greasier alternatives such as petrolatum or Aquaphor cream. Emollients aid in hydrating and softening psoriatic plaques. It is found to be effective in reducing itching, soreness, redness, and the extension of plaques in approximately 35 percent of patients [152]. It works by possibly reversing the effects of inflammation on the outermost layer of the epidermis [43,153].	There is a lack of receptivity due to cosmetic reasons where the cream causes stickiness or shininess on applied areas. Moreover, impracticability in less mobile patients—the elderly, who are not able to apply the emollients independently—makes it challenging to ensure effective treatment adherence [43].
Corticosteroids	Most frequently prescribed therapy able to cater to a wide range of disease severities as potency ranges from mild to ultra-potent [43,44,154]. Review by Castela et al. found that topical corticosteroids were efficacious and led to a more than 50 percent improvement in initial mild-to-severe psoriasis patients for at least 30 percent of patients [155]. Low cost, fast acting, and no skin irritation has increased patient receptivity for this therapeutic.	It cannot be used in the long-term management of psoriasis due to its more prominent side effects such as skin atrophy and striae, which are irreversible [156].
Coal Tar	Efficacious in clearing psoriatic plaques and is often used in combinatorial therapy with ultraviolet B (UVB) irradiation phototherapy [157,158].	It has an unpleasant odour and has a tendency to cause skin irritation even when used at low concentrations. It might also induce and cause skin cancer in rare cases [159].
Keratolytic Agents	Ointments that typically consist of 2–10 percent salicylic acid. These agents are used to soften psoriatic plaques and facilitate its removal. It is often used in combination with topical corticosteroids or coal tar to increase the effectiveness of treatment [43].	It is an irritant and can cause eye contamination if care is not exercised when used [43].
Anthralin or Dithranol	Another widely used treatment method, it is a antipsoriatic agent that is effective in targeting plaque-type psoriasis [43,44,160]. This treatment is commonly used in the Ingram regime, which comprises a daily coal tar bath, UVB phototherapy, and 24-hour application of an anthralin paste containing salicylic acid to prevent the oxidation of anthralin to inactive compounds [161].	When anthralin is oxidised, it causes staining of skin and clothes and is highly irritant due to the formation of free-radicals [162]. This topical treatment is not ideal for patients with inverse psoriasis in particular, as its highly irritant nature means applying it to sensitive parts of the body should be avoided.
Calcipotriene	A topical ointment that consists of vitamin D_3_ analogues that is safe and effective in treating mild-to-moderate psoriatic plaques [163]. Studies have elucidated that the efficacy of this treatment is similar to a medium-potency topical corticosteroid [164,165]. Additionally, its effectiveness is increased when used as part of a combinatorial therapy [32].	It is a mild irritant and has been found to cause adverse local effects in 15 percent of patients [166]. Furthermore, side effects such as potential toxicities and rate of relapse when calcipotriene is used for long-term management of psoriasis are still unknown.

**Table 3:** Overview of systemic treatments in the management of psoriasis.

Type of Therapy	Description and Mechanism of Action	Side Effects or Disadvantages
Phototherapy (UVB irradiation)	Ultraviolet B (UVB) irradiation uses wavelengths within the 300 to 320 nm range. It is one of the oldest treatments for moderate-to-severe psoriasis. It is often used in combinatorial therapy with coal tar and is effective in achieving remission in approximately 80 percent of patients after two to three weeks of intensive treatment [167]. Further, the risk of skin cancer associated with UVB therapy is relatively low [159,167]. Hence, this treatment is highly advantageous with its favourable therapeutic index and ability to be administered on an outpatient basis.	For it to be effective, it would require at least 30 treatments, and oftentimes patients find the coal tar unpleasant due to its smell. Additionally, its high cost and the time commitment required due to multiple treatments are notable disadvantages [43].
Photochemotherapy	Photochemotherapy, also known as PUVA, combines the photosensitising drug methoxsalen with ultraviolet A (UVA) phototherapy (320 to 400 nm). This treatment is highly efficacious, with over 85 percent of patients achieving lesion clearance after 20 to 30 sessions [168]. The mechanism of this therapeutic include the intercalation of methoxsalen into DNA, disrupting DNA synthesis and cell proliferation, and the suppression of cell-mediated immune responses in the skin [169]. The ease of treatment, high effectiveness, and no need for additional topical medication makes this a favourable therapeutic.	Maintenance therapy is required to prevent remission [170]. Short-term side effects are nausea, burning, and pruritus. While PUVA therapy is generally well tolerated, it carries the risk of long-term side effects, including an increased incidence of skin cancer, particularly squamous-cell carcinoma [171]. The therapy can also lead to photodamage, such as wrinkling, irregular pigmentation, and benign or premalignant keratoses. The risk of these side effects increases with the number of treatments, making it advisable to limit cumulative exposure to fewer than 160 sessions [172].
Methotrexate	Methotrexate, a folic acid antagonist, is a systemic treatment for severe psoriasis, particularly when phototherapy has been ineffective. It works by inhibiting DNA synthesis and cell proliferation, affecting rapidly dividing cells like those in psoriatic lesions [173]. Methotrexate is typically administered orally in three doses taken 12 hours apart once weekly or as a single weekly dose [174,175,176].	Although effective, methotrexate has significant side effects, particularly hepatotoxicity, leading to cirrhosis in some patients. Regular liver biopsies are recommended for long-term users to monitor for liver damage. The risk of cirrhosis increases with higher cumulative doses of the drug, especially above 1.5 g [177].
Cyclosporine	Cyclosporine is an immunosuppressive drug used to manage severe psoriasis that does not respond to other treatment methods. It works by inhibiting a calcineurin-dependent factor needed for T-cell proliferation, leading to rapid improvement in symptoms, usually within two weeks [178,179]. Low doses (3 to 5 mg/kg/day) are effective for more than 60% of patients [180].	Higher dosages increase the risk of adverse effects [180]. Cyclosporine is associated with adverse side effects such as hypertension and potentially irreversible renal damage [181,182]. The immunosuppressive properties might also bring about an increased risk of cancer. Treatment should generally not exceed one year due to its side effects, but most patients relapse within two to four months after stopping the treatment [180,183].
Systemic Retinoids (Etretinate)	Etretinate, a retinoid derivative of vitamin A, is used in the treatment of psoriasis by promoting epithelial differentiation and inhibiting malignant transformation [184]. Although its efficacy as a monotherapy is limited, etretinate is particularly beneficial in patients with erythrodermic, acral-localised, or generalised pustular psoriasis [185,186,187,188]. It is often combined with PUVA to enhance therapeutic outcomes [189,190,191].	Side effects are dose-dependent and include skin dryness, scaling, and erythema. Etretinate is highly teratogenic, and its long-lasting presence in the body—up to two years after discontinuation—requires stringent contraceptive measures in women of childbearing age [192]. Additionally, long-term use can lead to skeletal abnormalities, although the clinical significance of these changes is still unclear [193].
Systemic Corticosteroids	Oral corticosteroids are effective in controlling psoriasis and are typically reserved for short-term use in acutely ill patients with erythrodermic psoriasis [43].	These are generally avoided due to their significant side effects, such as Cushing’s syndrome, with prolonged use and the potential for severe disease exacerbation upon withdrawal. Furthermore, long-term usage can lead to the development of treatment-resistant forms of the disease [43].
Etanercept	Subcutaneous administration of a recombinant human TNF receptor fusion protein that binds and neutralises soluble TNF-α [194].	Etanercept acts as a decoy receptor, binding to TNF-α to prevent interactions with TNF receptors on cell surfaces. This inhibits the downstream inflammatory cascade, which reduces keratinocyte hyperproliferation and inflammation in psoriasis [194].
Infliximab	TNF-α-targeting chimeric monoclonal antibody, which is administered intravenously [195].	Infliximab binds and neutralises both soluble and transmembrane TNF-α. This blocks TNF-α-mediated inflammation, reducing inflammatory response and improving psoriatic lesions [195].
Adalimumab	Subcutaneously administered fully human monoclonal antibody which targets TNF-α [196].	Adalimumab binds soluble and membrane-bound TNF-α to block interaction with TNF receptors. This reduces pro-inflammatory cytokines production such as IL-1 and IL-6. This alleviates inflammation and associated symptoms [196].
Secukinumab	Subcutaneously administered human monoclonal antibody that targets IL-17A [197].	Secukinumab neutralises IL-17A to inhibit it interacting with its receptor. This reduces keratinocyte activation and associated inflammation [197].
Ixekizumab	Subcutaneously administered humanised monoclonal antibody that targets IL-17A [198].	Ixekizumab binds to IL-17A and blocks its interaction with IL-17RA. This inhibits IL-17A-mediated signalling pathways, reducing psoriatic inflammation and keratinocyte hyperproliferation [198].
Guselkumab	Subcutaneously administered human monoclonal antibody which targets IL-23 [199].	Guselkumab binds to the p19 subunit of IL-23 to inhibit its interaction with IL-23 receptor. This blocks the activation of Th17 cells and downstream cytokine production such as IL-17 and IL-22 [199].
Risankizumab	Subcutaneously administered humanised monoclonal antibody that targets IL-23 [200].	Risankizumab inhibits IL-23 by binding to its p19 subunit. This blocks downstream signalling pathways linked with Th17 differentiation. This reduces inflammation and keratinocyte hyperproliferation [200].
Ustekinumab	Subcutaneously administered fully human monoclonal antibody which targets the IL-12 and IL-23 [201].	Ustekinumab binds the p40 subunit in IL-12 and IL-23 to block interactions with receptors of T cells and natural killer cells. This inhibits the activation of Th1 and Th17 cells, which reduces inflammation and keratinocyte hyperproliferation [201].
Tildrakizumab	Subcutaneously administered humanised monoclonal antibody which targets IL-23 [202].	Tildrakizumab binds to the p19 subunit of IL-23. This blocks IL-23 from activating its receptor, thus reducing the differentiation and proliferation of Th17 cells. This results in a decrease in inflammation and keratinocyte hyperproliferation [202].

**Table 4:** Overview of alternative treatments employed in the management of psoriasis.

Type of Therapy	Description And Mechanism Of Action	Side Effects or Disadvantages
Climatotherapy	This treatment involves travel to a specified location to use climatic factors for healing. A well-established example is the Dead Sea in Israel, where the high salinity and mineral concentrations of the water is coupled with natural forms of UV-A/B phototherapy as patients are highly exposed to the sun’s UV rays in a low-altitude area [203].	It has been suggested that the ethnicities of the patients should be considered when referring for Dead Sea climatotherapy, as a study using Israeli patients found that the treatment could give rise to chronic solar damage side effects such as wrinkles [204], whereas in a Danish cohort, the risk of non-melanoma skin cancer was increased almost 5-fold compared to the Israeli population [205].
Balneotherapy	Also referred to as spa therapy, balneotherapy is a form of hydrotherapy and refers to treatment by bathing in mineral water. An example is Leopoldine spa water in Tuscany, Italy, where the water is hypotonic and rich in sulphate. After 4 weeks of treatment, an improvement of PASI score was seen alongside a significant decrease in epidermal CD4+ and CD8+ lymphocytes [206].	There do not seem to be many reported side effects outside of mild skin irritation; however, there is a financial burden due to travel to the spas. This, coupled with the long duration of treatments, limits the widespread use of balneotherapy [207].

**Table 5:** Emerging therapeutics in development for psoriasis management.

Name	Description	Mechanism of Action
SGX302	Phototherapy using wavelengths within the visible region to activate synthetically manufactured hypericin, which is applied as an ointment [208].	Hypericin is absorbed by cells at the area where it is topically applied. After it is activated by fluorescent light, it forms oxygen radicals that lead to cellular toxicity, thus killing targeted cells. Destruction of targeted cells may facilitate the clearing of psoriatic lesions [208].
EDP1815	A non-live strain of *Prevotella histicola* that is administered orally and is gut restricted. It is a novel immunomodulatory therapeutic class targeting the small intestine to produce systemic anti-inflammatory responses [209].	EDP1815 leverages the anti-inflammatory functions of the small intestine to reduce inflammation in other parts of the body. This process involves three steps: (1) bacterial components interact with pattern recognition receptors on gut immune cells such as dendritic cells, (2) these interactions in the mesenteric lymph nodes generate regulatory CD4+ T cells, and (3) these T cells then circulate to distant sites to reduce inflammation [210,211].
Roflumilast Cream(ARQ-151)	A PDE-4 inhibitor to be applied topically once daily. The cream comprises roflumilast in a high-water-content moisturising cream, which also contains the cosmetic solvent ethoxydiglycol [212].	Roflumilast inhibits PDE-4, which is an enzyme that is more active in psoriatic lesions [213]. This inhibition increases cAMP levels [214], which results in a reduction in key inflammatory mediators like TNF-α, IFN-γ, IL-17, and IL-23, which helps reduce inflammation [215].
HB0017	A recombinant humanised IgG1 monoclonal antibody that is able to target human IL-17A with high specificity and affinity [216].	HB0017 binds to IL-17A with high specificity and affinity at the physiological interface with IL-17RA. It then effectively antagonises the functions of IL-17A to inhibit psoriasis-like inflammation. This may facilitate reduction in IL-17-induced inflammation [216,217].
GN-037	Topical cream comprising four active compounds—clobetasol 17-propionate, salicylic acid, urea, and retinoic acid [218].	The four active pharmaceutical compounds target various aspects of psoriasis. This includes reducing inflammation, promoting skin renewal, moisturising, and preventing skin discoloration [218].
IBI112	IBI112 is a humanised anti-IL23p19 monoclonal antibody developed with hybridoma technology. It is first created by immunising mice with a mouse IL12p40/human IL23p19 complex. Thereafter, clone 17D1 with high specificity for the IL-23p19 subunit is selected. IBI112 is then formed through enhancing 17D1 with mutations in the Fc region [219].	IBI112 targets the IL-23/IL-17 axis, which is involved in the pathogenesis of psoriasis. By neutralising the IL23p19 subunit of IL-23, IBI112 blocks IL-23 from binding to its receptor, which subsequently inhibits downstream STAT3 phosphorylation. This reduces IL-17 production, which effectively decreases inflammation and skin thickness [219].
SAR441566	Orally administered therapeutic agent, which is an inhibitor of the TNF cytokine [220,221].	SAR441566 acts by stabilising the asymmetrical form of the soluble TNF trimer. This prevents TNF from interacting with the TNFR1 receptor. This inhibition blocks the production of pro-inflammatory cytokines and facilitates reduction in inflammation associated with psoriasis [221]

## Data Availability

Not applicable.

## Abbreviations

Antigen-presenting cells (APCs), non-coding RNAs (ncRNAs), microRNA (miRNAs), primary miRNA (Pri-miRNA), precursor miRNA (Pre-miRNA), argonaute protein (Ago), RNA-induced silencing complex (RISC), antimicrobial peptides (AMPs), plasmacytoid dendritic cells (pDCs), Toll-like receptor 9 (TLR9), myeloid dendritic cells (mDCs), nuclear factor kappa-light-chain-enhancer of activated B cells (NF-κB), progression protein phosphatase 6 (ppp6c), tissue inhibitor of matrix metalloproteinase 3 (TIMP-3), tumour necrosis factor-α (TNF-α), TNF-α-converting enzyme (TACE), signal transducer and activator of transcription 3 (STAT3), transforming growth factor-β (TGF-β), protein 63 (p63), suppressor of cytokine signalling 3 (SOCS-3), quantitative real-time PCR (qRT-PCR), maternally expressed gene 3 (MEG3), long non-coding RNAs (lncRNAs), framework nucleic acids (FNAs), psoriasis area and severity index (PASI), fibroblast growth factor receptor 2 (FGFR2).
